# Supplementary material for: Host body size, not host population size, predicts genome-wide effective population size of parasites
Source: Evol Lett. 2023 Jun 5;7(4):285–92. doi: 10.1093/evlett/qrad026 (PMC10355176; doi:10.1093/evlett/qrad026)

**Fig S1. Cladogram of *Columbicola* host species used for the cophylogenetic analyses. The tree file can be found in (reserved DOI: 10.6084/m9.figshare.21269640; private link for review: https://figshare.com/s/2f2de5dc909155da815a)**

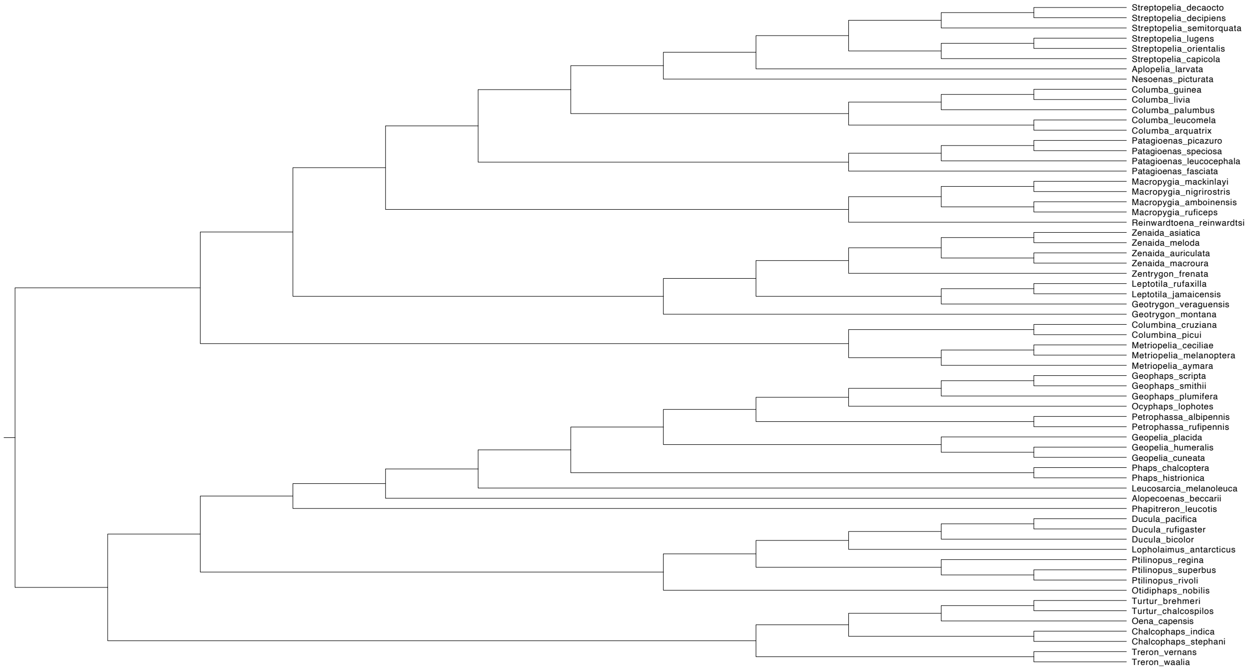

**Fig S2. Time-calibrated tree of *Columbicola* used for the phylogenetic comparative analyses. Numbers on branches are ultrafast bootstrap values from IQ-TREE. The full tree can be found in (reserved DOI: 10.6084/m9.figshare.21269640; private link for review: https://figshare.com/s/2f2de5dc909155da815a)**

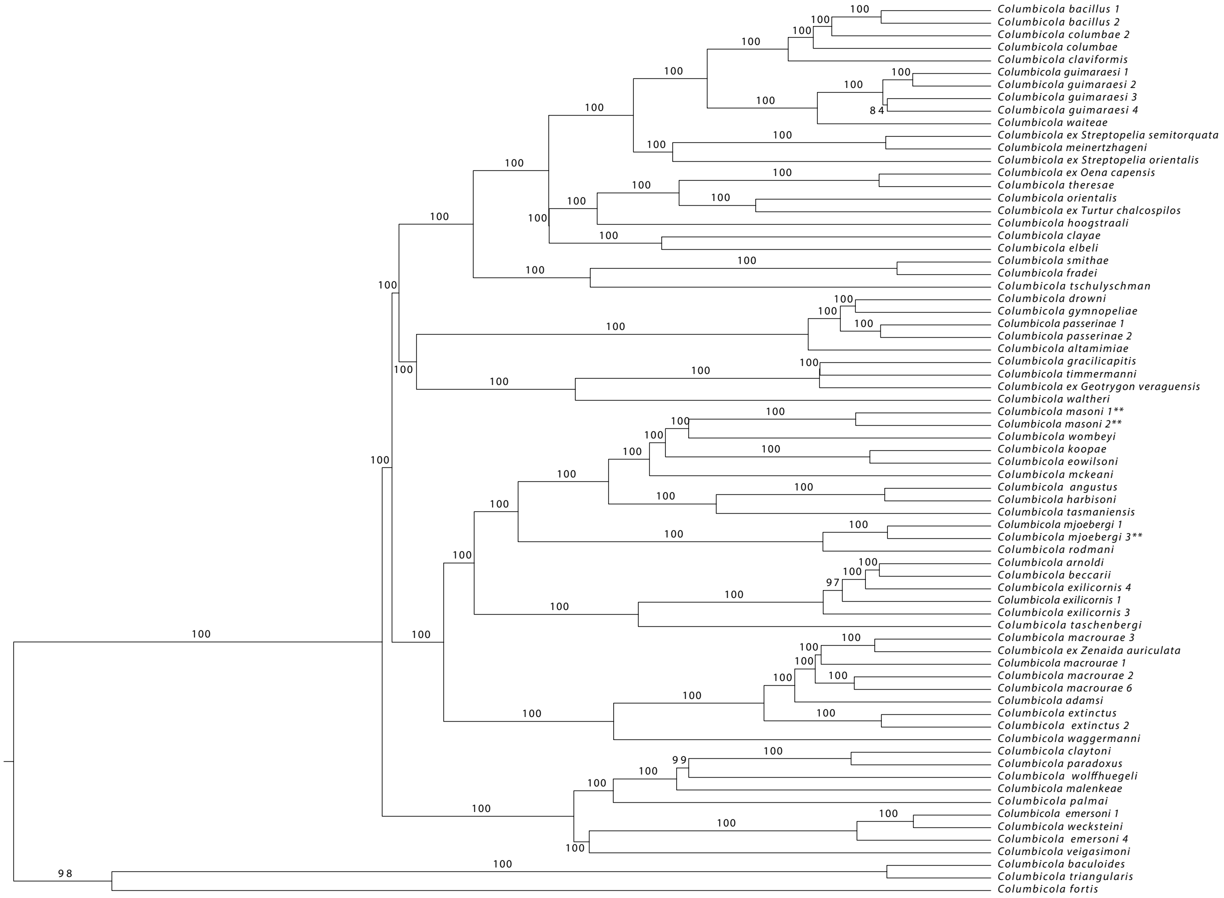

**Fig S3. Scatter plots showing the relationship between theta and host population size (both untransformed and log transformed).**

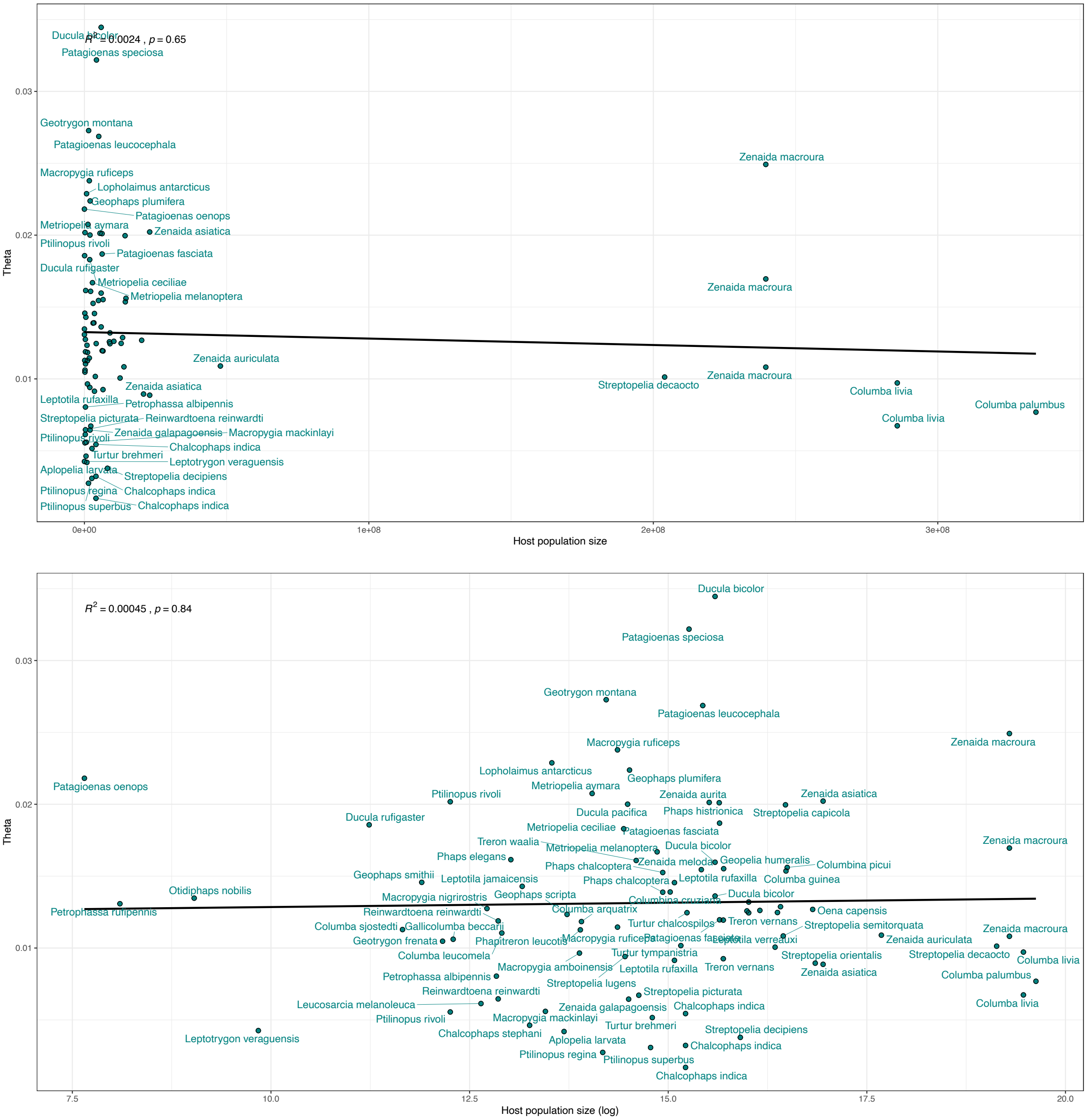

Supplement: qrad026_suppl_Supplementary_Figures [file qrad026_suppl_supplementary_figures.pdf]
